# Bis(2,3-diamino­pyridinium) phthalate dihydrate

**DOI:** 10.1107/S1600536811055838

**Published:** 2012-01-14

**Authors:** Madhukar Hemamalini, Jia Hao Goh, Hoong-Kun Fun

**Affiliations:** a X-ray Crystallography Unit, School of Physics, Universiti Sains Malaysia, 11800 USM, Penang, Malaysia

## Abstract

The complete anion of the title hydrated mol­ecular salt, 2C_5_H_8_N_3_^+^·C_8_H_4_O_4_^−^·2H_2_O, is generated by a crystallographic twofold axis. In the crystal, the cations, anions and water mol­ecules are connected by N—H⋯O, O—H⋯O and C—H⋯O hydrogen bonds, forming a three-dimensional network. The crystal structure also features C—H⋯π inter­actions.

## Related literature

For background to hydrogen-bonding patterns of 2-amino­pyridine derivatives, see: Gellert & Hsu (1988 ▶); Banerjee & Murugavel (2004 ▶). For related structures, see: Hemamalini & Fun (2010*a* ▶,*b* ▶,*c* ▶). For the stability of the temperature controller used in the data collection, see: Cosier & Glazer (1986 ▶).
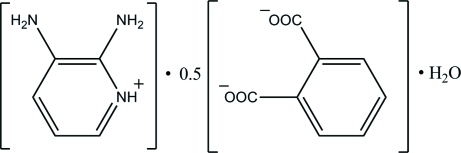


## Experimental

### 

#### Crystal data

2C_5_H_8_N_3_^+^·C_8_H_4_O_4_^−^·2H_2_O
*M* *_r_* = 420.43
Monoclinic, 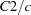

*a* = 15.795 (5) Å
*b* = 13.083 (4) Å
*c* = 11.012 (4) Å
β = 115.194 (5)°
*V* = 2059.2 (11) Å^3^
*Z* = 4
Mo *K*α radiation
μ = 0.10 mm^−1^
*T* = 100 K
0.42 × 0.36 × 0.03 mm

#### Data collection

Bruker APEXII DUO CCD diffractometer
Absorption correction: multi-scan (*SADABS*; Bruker, 2009 ▶) *T*_min_ = 0.958, *T*_max_ = 0.997
10343 measured reflections
2955 independent reflections
2148 reflections with *I* > 2σ(*I*)
*R*_int_ = 0.042

#### Refinement

*R*[*F* ^2^ > 2σ(*F* ^2^)] = 0.043
*wR*(*F* ^2^) = 0.125
*S* = 1.04
2955 reflections
184 parameters
All H-atom parameters refined
Δρ_max_ = 0.33 e Å^−3^
Δρ_min_ = −0.26 e Å^−3^

### 

Data collection: *APEX2* (Bruker, 2009 ▶); cell refinement: *SAINT* (Bruker, 2009 ▶); data reduction: *SAINT*; program(s) used to solve structure: *SHELXTL* (Sheldrick, 2008 ▶); program(s) used to refine structure: *SHELXTL*; molecular graphics: *SHELXTL*; software used to prepare material for publication: *SHELXTL* and *PLATON* (Spek, 2009 ▶).

## Supplementary Material

Crystal structure: contains datablock(s) global, I. DOI: 10.1107/S1600536811055838/hb6574sup1.cif

Structure factors: contains datablock(s) I. DOI: 10.1107/S1600536811055838/hb6574Isup2.hkl

Supplementary material file. DOI: 10.1107/S1600536811055838/hb6574Isup3.cml

Additional supplementary materials:  crystallographic information; 3D view; checkCIF report

## Figures and Tables

**Table 1:** Hydrogen-bond geometry (Å, °) *Cg*1 and *Cg*2 are the centroids of the C6–C8/C6A–C8A and N1/C1–C5 rings, respectively.

*D*—H⋯*A*	*D*—H	H⋯*A*	*D*⋯*A*	*D*—H⋯*A*
N2—H5⋯O2^i^	0.90 (2)	2.03 (2)	2.9155 (19)	169.0 (18)
N1—H6⋯O2^ii^	0.98 (2)	1.790 (19)	2.7565 (18)	170.3 (19)
N2—H7⋯O1^ii^	0.92 (2)	1.99 (2)	2.8976 (19)	168.1 (19)
N3—H9⋯O1*W*^iii^	0.89 (2)	2.09 (2)	2.978 (2)	179 (3)
N3—H10⋯O2^i^	0.89 (2)	2.23 (2)	3.078 (2)	158.8 (17)
O1*W*—H11⋯O1^iv^	0.95 (3)	1.86 (3)	2.7878 (18)	165 (3)
O1*W*—H12⋯O1^v^	0.92 (2)	1.99 (2)	2.8641 (18)	158.2 (19)
C3—H2⋯O1*W*^vi^	0.984 (17)	2.516 (18)	3.369 (2)	145.0 (14)
C4—H8⋯*Cg*1^iii^	1.01 (2)	2.76 (2)	3.629 (2)	144.3 (15)
C6—H3⋯*Cg*2^vii^	0.959 (18)	2.568 (18)	3.497 (2)	163.4 (14)

Symmetry codes: (i) 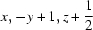; (ii) 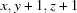; (iii) 
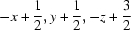
; (iv) 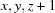; (v) 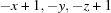; (vi) 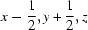; (vii) 
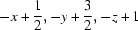
.

**Fig. 1.**
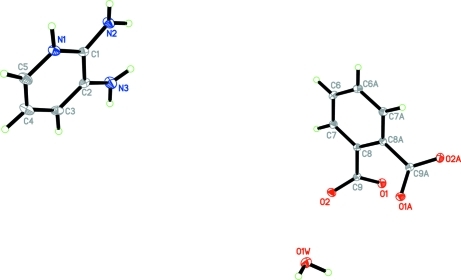
The molecule of (I) with 30% probability displacement ellipsoids. Atoms labelled A are generated by -x+1, y, -z+1/2.

**Fig. 2.**
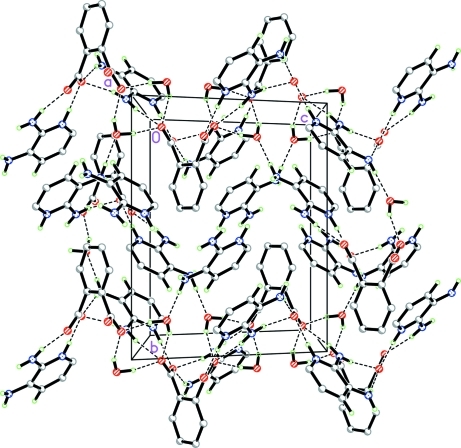
The crystal packing of the title compound showing hydrogen-bonded (dashed lines) networks. H atoms not involved in hydrogen bond interactions are omitted for clarity.

## supplementary crystallographic information

### Comment

2-Aminopyridine and its derivatives are amomg the most frequently used synthons in supramolecular chemistry based on hydrogen bonds (Gellert & Hsu, 1988; Banerjee & Murugavel, 2004). A series of similar complexes formed from 2-aminopyridine and carboxylates has been reported previously (Hemamalini & Fun, 2010*a,b,c*). The present work, 2,3-diaminopyridinium phthalate dihydrate, (2/1/1) (I), is a continuation of our structural study of complexes of the 2,3-diaminopyridinium system.

The asymmetric unit of the title compound, (I), contains one 2,3-diamino- pyridinium cation, a half of phthalate anion (which lies on a twofold axis; -x+1, y, -z+1/2) and a water molecule as shown in Fig. 1. The dihedral angle between the pyridine (N1/C1–C5) and benzene (C6–C8/ C6A–C8A) ring is 80.61 (7)°.

In the crystal structure (Fig. 2), the ion pairs and water molecules are connected *via* N—H···O, O—H···O and C—H···O (Table 1) hydrogen bonds forming a three-dimensional network. The crystal structure is further stabilized by C—H···π interactions involving the centroids of the C6–C8/C6A–C8A (Cg1) and N1/C1–C5 (Cg2) rings.

### Experimental

A hot methanol solution (20 ml) of 2,3-diaminopyridine (27 mg, Aldrich) and phthalic acid (42 mg, Merck) were mixed and warmed over a magnetic stirrer hotplate for a few minutes. The resulting solution was allowed to cool slowly at room temperature and brown plates of the title compound appeared after a few days.

### Refinement

All hydrogen atoms were located from a difference Fourier maps and refined freely [N–H = 0.89 (2)–0.98 (2)Å; O–H = 0.91 (2)– 0.95 (3) Å and C–H = 0.959 (18)–1.01 (2) Å]. The highest residual electron density peak is located at 0.73 Å from C9 and the deepest hole is located at 0.78 Å from C9.

### Crystal data

**Table:**

2C_5_H_8_N_3_^+^·C_8_H_4_O_4_^−^·2H_2_O	*F*(000) = 888
*M**_r_* = 420.43	*D*_x_ = 1.356 Mg m^−^^3^
Monoclinic, *C*2/*c*	Mo *K*α radiation, λ = 0.71073 Å
Hall symbol: -C 2yc	Cell parameters from 3390 reflections
*a* = 15.795 (5) Å	θ = 2.8–29.8°
*b* = 13.083 (4) Å	µ = 0.10 mm^−^^1^
*c* = 11.012 (4) Å	*T* = 100 K
β = 115.194 (5)°	Plate, brown
*V* = 2059.2 (11) Å^3^	0.42 × 0.36 × 0.03 mm
*Z* = 4	

### Data collection

**Table:**

Bruker APEXII DUO CCD diffractometer	2955 independent reflections
Radiation source: fine-focus sealed tube	2148 reflections with *I* > 2σ(*I*)
graphite	*R*_int_ = 0.042
φ and ω scans	θ_max_ = 29.9°, θ_min_ = 2.1°
Absorption correction: multi-scan (*SADABS*; Bruker, 2009)	*h* = −22→22
*T*_min_ = 0.958, *T*_max_ = 0.997	*k* = −18→18
10343 measured reflections	*l* = −14→14

### Refinement

**Table:**

Refinement on *F*^2^	Primary atom site location: structure-invariant direct methods
Least-squares matrix: full	Secondary atom site location: difference Fourier map
*R*[*F*^2^ > 2σ(*F*^2^)] = 0.043	Hydrogen site location: inferred from neighbouring sites
*wR*(*F*^2^) = 0.125	All H-atom parameters refined
*S* = 1.03	*w* = 1/[σ^2^(*F*_o_^2^) + (0.0653*P*)^2^ + 0.7423*P*] where *P* = (*F*_o_^2^ + 2*F*_c_^2^)/3
2955 reflections	(Δ/σ)_max_ < 0.001
184 parameters	Δρ_max_ = 0.33 e Å^−^^3^
0 restraints	Δρ_min_ = −0.25 e Å^−^^3^

### Special details

**Table:**

Experimental. The crystal was placed in the cold stream of an Oxford Cryosystems Cobra open-flow nitrogen cryostat (Cosier & Glazer, 1986) operating at 100.0 (1) K.
Geometry. All s.u.'s (except the s.u. in the dihedral angle between two l.s. planes) are estimated using the full covariance matrix. The cell s.u.'s are taken into account individually in the estimation of s.u.'s in distances, angles and torsion angles; correlations between s.u.'s in cell parameters are only used when they are defined by crystal symmetry. An approximate (isotropic) treatment of cell s.u.'s is used for estimating s.u.'s involving l.s. planes.
Refinement. Refinement of F^2^ against ALL reflections. The weighted R-factor wR and goodness of fit S are based on F^2^, conventional R-factors R are based on F, with F set to zero for negative F^2^. The threshold expression of F^2^ > 2σ(F^2^) is used only for calculating R-factors(gt) etc. and is not relevant to the choice of reflections for refinement. R-factors based on F^2^ are statistically about twice as large as those based on F, and R- factors based on ALL data will be even larger.

### Fractional atomic coordinates and isotropic or equivalent isotropic displacement parameters (Å^2^)

**Table:**

	*x*	*y*	*z*	*U*_iso_*/*U*_eq_	
N1	0.23077 (8)	0.94380 (9)	1.03746 (11)	0.0227 (3)	
N2	0.30262 (8)	0.94315 (10)	0.89459 (13)	0.0256 (3)	
N3	0.17815 (10)	0.78666 (11)	0.74285 (14)	0.0330 (3)	
C1	0.23819 (9)	0.90400 (10)	0.93023 (13)	0.0192 (3)	
C2	0.17516 (9)	0.82430 (9)	0.85688 (13)	0.0210 (3)	
C3	0.11106 (9)	0.79131 (11)	0.90331 (14)	0.0249 (3)	
C4	0.10585 (11)	0.83570 (13)	1.01540 (15)	0.0313 (3)	
C5	0.16621 (11)	0.91224 (13)	1.08149 (15)	0.0310 (3)	
O1	0.43751 (6)	0.07071 (7)	0.10193 (9)	0.0225 (2)	
O2	0.32273 (6)	0.12167 (7)	0.15491 (9)	0.0225 (2)	
C6	0.45346 (10)	0.41720 (10)	0.20469 (14)	0.0242 (3)	
C7	0.40590 (9)	0.32537 (10)	0.16012 (13)	0.0201 (3)	
C8	0.45249 (8)	0.23274 (9)	0.20552 (12)	0.0160 (2)	
C9	0.40094 (8)	0.13405 (9)	0.15149 (12)	0.0168 (2)	
O1W	0.45043 (8)	0.10881 (8)	0.86130 (12)	0.0315 (3)	
H1	0.3388 (12)	0.3242 (12)	0.0959 (17)	0.028 (4)*	
H2	0.0692 (11)	0.7346 (13)	0.8557 (17)	0.026 (4)*	
H3	0.4209 (12)	0.4807 (14)	0.1749 (17)	0.032 (4)*	
H4	0.1689 (13)	0.9496 (15)	1.160 (2)	0.043 (5)*	
H5	0.3138 (13)	0.9165 (14)	0.8275 (19)	0.033 (5)*	
H6	0.2697 (12)	1.0029 (15)	1.0806 (18)	0.036 (5)*	
H7	0.3468 (13)	0.9879 (15)	0.9506 (19)	0.039 (5)*	
H8	0.0557 (13)	0.8149 (14)	1.044 (2)	0.042 (5)*	
H9	0.1404 (14)	0.7328 (17)	0.712 (2)	0.046 (5)*	
H10	0.2265 (14)	0.7967 (14)	0.723 (2)	0.040 (5)*	
H11	0.4555 (16)	0.1035 (17)	0.950 (3)	0.064 (7)*	
H12	0.4828 (16)	0.0542 (18)	0.850 (2)	0.062 (7)*	

### Atomic displacement parameters (Å^2^)

**Table:**

	*U*^11^	*U*^22^	*U*^33^	*U*^12^	*U*^13^	*U*^23^
N1	0.0235 (6)	0.0244 (6)	0.0181 (5)	−0.0068 (5)	0.0070 (4)	−0.0020 (4)
N2	0.0237 (6)	0.0281 (6)	0.0260 (6)	−0.0104 (5)	0.0116 (5)	−0.0096 (5)
N3	0.0323 (7)	0.0332 (7)	0.0364 (7)	−0.0153 (6)	0.0174 (6)	−0.0188 (6)
C1	0.0186 (6)	0.0182 (6)	0.0175 (6)	−0.0001 (4)	0.0043 (5)	0.0016 (4)
C2	0.0195 (6)	0.0181 (6)	0.0197 (6)	−0.0001 (5)	0.0028 (5)	0.0004 (5)
C3	0.0232 (6)	0.0223 (6)	0.0224 (6)	−0.0060 (5)	0.0033 (5)	0.0024 (5)
C4	0.0312 (7)	0.0392 (8)	0.0237 (7)	−0.0138 (6)	0.0119 (6)	0.0001 (6)
C5	0.0348 (8)	0.0393 (8)	0.0223 (7)	−0.0128 (6)	0.0153 (6)	−0.0043 (6)
O1	0.0229 (5)	0.0220 (5)	0.0222 (5)	−0.0006 (4)	0.0093 (4)	−0.0059 (4)
O2	0.0205 (4)	0.0227 (5)	0.0253 (5)	−0.0041 (4)	0.0106 (4)	−0.0029 (4)
C6	0.0252 (7)	0.0173 (6)	0.0297 (7)	0.0038 (5)	0.0113 (6)	0.0033 (5)
C7	0.0191 (6)	0.0200 (6)	0.0196 (6)	0.0017 (5)	0.0068 (5)	0.0022 (4)
C8	0.0180 (6)	0.0171 (6)	0.0140 (5)	−0.0005 (4)	0.0078 (4)	−0.0002 (4)
C9	0.0178 (5)	0.0173 (6)	0.0124 (5)	0.0007 (4)	0.0036 (4)	0.0006 (4)
O1W	0.0361 (6)	0.0276 (6)	0.0363 (6)	0.0102 (4)	0.0208 (5)	0.0120 (4)

### Geometric parameters (Å, °)

**Table:**

N1—C1	1.3404 (17)	C4—H8	1.01 (2)
N1—C5	1.3663 (18)	C5—H4	0.98 (2)
N1—H6	0.98 (2)	O1—C9	1.2596 (15)
N2—C1	1.3388 (17)	O2—C9	1.2623 (15)
N2—H5	0.899 (19)	C6—C6^i^	1.382 (3)
N2—H7	0.92 (2)	C6—C7	1.3910 (19)
N3—C2	1.3683 (19)	C6—H3	0.959 (18)
N3—H9	0.89 (2)	C7—C8	1.3954 (17)
N3—H10	0.89 (2)	C7—H1	0.993 (17)
C1—C2	1.4304 (18)	C8—C8^i^	1.400 (2)
C2—C3	1.383 (2)	C8—C9	1.5072 (17)
C3—C4	1.398 (2)	O1W—H11	0.95 (3)
C3—H2	0.983 (17)	O1W—H12	0.91 (2)
C4—C5	1.360 (2)		
			
C1—N1—C5	123.61 (12)	C5—C4—H8	119.7 (11)
C1—N1—H6	117.6 (11)	C3—C4—H8	121.0 (11)
C5—N1—H6	118.5 (10)	C4—C5—N1	119.44 (14)
C1—N2—H5	122.2 (12)	C4—C5—H4	126.7 (11)
C1—N2—H7	120.2 (11)	N1—C5—H4	113.8 (11)
H5—N2—H7	116.0 (16)	C6^i^—C6—C7	120.25 (8)
C2—N3—H9	111.0 (13)	C6^i^—C6—H3	119.9 (10)
C2—N3—H10	122.9 (13)	C7—C6—H3	119.8 (10)
H9—N3—H10	121.6 (18)	C6—C7—C8	120.03 (12)
N2—C1—N1	118.16 (12)	C6—C7—H1	121.1 (10)
N2—C1—C2	123.13 (12)	C8—C7—H1	118.9 (10)
N1—C1—C2	118.67 (12)	C7—C8—C8^i^	119.70 (7)
N3—C2—C3	122.93 (12)	C7—C8—C9	119.26 (11)
N3—C2—C1	119.54 (12)	C8^i^—C8—C9	120.95 (6)
C3—C2—C1	117.49 (12)	O1—C9—O2	124.30 (11)
C2—C3—C4	121.61 (13)	O1—C9—C8	117.67 (11)
C2—C3—H2	118.0 (10)	O2—C9—C8	117.99 (11)
C4—C3—H2	120.4 (10)	H11—O1W—H12	105.6 (19)
C5—C4—C3	119.14 (13)		
			
C5—N1—C1—N2	178.22 (13)	C3—C4—C5—N1	−0.3 (2)
C5—N1—C1—C2	0.2 (2)	C1—N1—C5—C4	0.6 (2)
N2—C1—C2—N3	−1.5 (2)	C6^i^—C6—C7—C8	−0.9 (2)
N1—C1—C2—N3	176.36 (12)	C6—C7—C8—C8^i^	−0.9 (2)
N2—C1—C2—C3	−179.27 (13)	C6—C7—C8—C9	−177.35 (12)
N1—C1—C2—C3	−1.39 (18)	C7—C8—C9—O1	126.40 (13)
N3—C2—C3—C4	−175.91 (14)	C8^i^—C8—C9—O1	−50.01 (19)
C1—C2—C3—C4	1.8 (2)	C7—C8—C9—O2	−51.38 (16)
C2—C3—C4—C5	−0.9 (2)	C8^i^—C8—C9—O2	132.22 (15)

Symmetry codes: (i) −*x*+1, *y*, −*z*+1/2.

### Hydrogen-bond geometry (Å, °)

**Table:**

Cg1 and Cg2 are the centroids of the C6–C8/C6A–C8A and N1/C1–C5 rings, respectively.

**Table:**

*D*—H···*A*	*D*—H	H···*A*	*D*···*A*	*D*—H···*A*
N2—H5···O2^ii^	0.90 (2)	2.03 (2)	2.9155 (19)	169.0 (18)
N1—H6···O2^iii^	0.98 (2)	1.790 (19)	2.7565 (18)	170.3 (19)
N2—H7···O1^iii^	0.92 (2)	1.99 (2)	2.8976 (19)	168.1 (19)
N3—H9···O1W^iv^	0.89 (2)	2.09 (2)	2.978 (2)	179 (3)
N3—H10···O2^ii^	0.89 (2)	2.23 (2)	3.078 (2)	158.8 (17)
O1W—H11···O1^v^	0.95 (3)	1.86 (3)	2.7878 (18)	165 (3)
O1W—H12···O1^vi^	0.92 (2)	1.99 (2)	2.8641 (18)	158.2 (19)
C3—H2···O1W^vii^	0.984 (17)	2.516 (18)	3.369 (2)	145.0 (14)
C4—H8···Cg1^iv^	1.01 (2)	2.76 (2)	3.629 (2)	144.3 (15)
C6—H3···Cg2^viii^	0.959 (18)	2.568 (18)	3.497 (2)	163.4 (14)

Symmetry codes: (ii) *x*, −*y*+1, *z*+1/2; (iii) *x*, *y*+1, *z*+1; (iv) −*x*+1/2, *y*+1/2, −*z*+3/2; (v) *x*, *y*, *z*+1; (vi) −*x*+1, −*y*, −*z*+1; (vii) *x*−1/2, *y*+1/2, *z*; (viii) −*x*+1/2, −*y*+3/2, −*z*+1.

## References

[bb1] Banerjee, S. & Murugavel, R. (2004). *Cryst. Growth Des.* **4**, 545–552.

[bb2] Bruker (2009). *APEX2*, *SAINT* and *SADABS* Bruker AXS Inc., Madison, Wisconsin, USA.

[bb3] Cosier, J. & Glazer, A. M. (1986). *J. Appl. Cryst.* **19**, 105–107.

[bb4] Gellert, R. W. & Hsu, I.-N. (1988). *Acta Cryst.* C**44**, 311–313. 10.1107/s0108270187009879 3271545

[bb5] Hemamalini, M. & Fun, H.-K. (2010*a*). *Acta Cryst.* E**66**, o1418–o1419. 10.1107/S1600536810018210 PMC2979380 21579496

[bb6] Hemamalini, M. & Fun, H.-K. (2010*b*). *Acta Cryst.* E**66**, o1480–o1481. 10.1107/S1600536810019239 PMC2979470 21579546

[bb7] Hemamalini, M. & Fun, H.-K. (2010*c*). *Acta Cryst.* E**66**, o1496–o1497. 10.1107/S1600536810019677 PMC2979552 21579558

[bb8] Sheldrick, G. M. (2008). *Acta Cryst.* A**64**, 112–122. 10.1107/S0108767307043930 18156677

[bb9] Spek, A. L. (2009). *Acta Cryst.* D**65**, 148–155. 10.1107/S090744490804362X PMC2631630 19171970

